# A Chromosome-Level Genome Assembly of the Parasitic Wasp *Chelonus formosanus* Sonan 1932 (Hymenoptera: Braconidae)

**DOI:** 10.1093/gbe/evac006

**Published:** 2022-01-28

**Authors:** Jian-Feng Liu, Hai-Yan Zhao, Yan-Fei Song, Yuan-Chan Yu, Mao-Fa Yang

**Affiliations:** 1 State Key Laboratory Breeding Base of Green Pesticide and Agricultural Bioengineering, Key Laboratory of Green Pesticide and Agricultural Bioengineering, Ministry of Education, Guizhou University, Guiyang, China; 2 Institute of Entomology, Guizhou University; Guizhou Provincial Key Laboratory for Agricultural Pest Management of the Mountainous Region; Scientific Observing and Experimental Station of Crop Pest in Guiyang, Ministry of Agriculture, Guiyang, China; 3 College of Tobacco Science, Guizhou University, Guiyang, China

**Keywords:** parasitic wasp, genome, comparative genomics, gene family evolution

## Abstract

*Chelonus formosanus* Sonan 1932 (Hymenoptera: Braconidae) is a wasp capable of parasitizing a variety of lepidopteran pests at the “egg-larval” stage which distributes throughout Taiwan, Guangdong, Zhejiang, and Hainan provinces of China. This wasp has been successfully used to control pests such as *Spodoptera litura* Fabricius, 1775, *Spodoptera frugiperda* (JE Smith, 1797), *Spodoptera exigua* (Hübner, 1808), and *Helicoverpa armigera* (Hübner, 1808). So far, there is only one genome assembled from the *Chelonus* genus [*Chelonus insularis* (Cresson, 1865)] and it is fragmented with 455 scaffolds. Here, we report a chromosome-level genome assembly of *C. formosanus*, which was sequenced using PacBio, Illumina, and Hi-C technologies. The long reads were 35.4 Gb (∼150× coverage) with an average length of 15.23 kb. The size of the genome assembly was 139.59 Mb. More than 99.46% of the assembled sequences were anchored to seven pseudochromosomes (138.84 Mb). The Benchmarking University Single-Copy Orthologs (BUSCO) assessment results showed 99.0% of the 1,367 genes (insect_odb10 database) were completely present. We annotated 11,242 protein-coding genes including 98.6% of BUSCO complete genes that were recovered. Nearly one-fourth of the genome assembly (22.25%) was annotated as repetitive sequences and 324 noncoding RNAs were predicted. There were 58 gene families found with significant expansion including allelopathic families (odorant receptors and ionotropic receptors), which may play a crucial role in efficiently locating a wide range of hosts. This high-quality genome assembly and annotation could provide a highly valuable resource of parasitic wasp for the biological control of Lepidoptera pest.

Significance

*Chelonus formosanus* is an important natural predator of agricultural pests and can parasitize a variety of lepidopteran pest species. However, at present, its genetic data are extremely limited. In order to understand the genetic background of *C. formosanus* more comprehensively, we sequenced its whole genome, which provided a highly valuable resource for understanding its parasitic potential and evolution.

## Introduction

The cosmopolitan genus *Chelonus* Panzer, 1806, harbors near 360 known species, which are ovo-larval endoparasitoids of Lepidoptera (Zhang et al. 2006). *Chelonus* normally regulate the metamorphic process to kill host larva during their final instar (Jones 1985; Zhang et al. 2006). The genus *Chelonus* have numerous associations with many *Spodoptera* species which include some important agricultural pests in the world (Jones 1985). So far, we found only one whole-genome assembly sequenced from the *Chelonus* genus (*Chelonus insularis* Cresson, 1865) was deposited on NCBI and it is fragmented with 455 scaffolds. To increase genomic resource from this insect genus and provide chromosomal information at the same time, we sequenced and assembled the whole genome of *Chelonus formosanus* Sonan using PacBio, Illumina, and Hi-C technologies. We also annotated protein-coding genes and analyzed the evolution of gene families across 16 species in different orders, including *C. formosanus* and *C. insularis*.

## Results and Discussion

### Genome Assembly

**Fig. 1.**
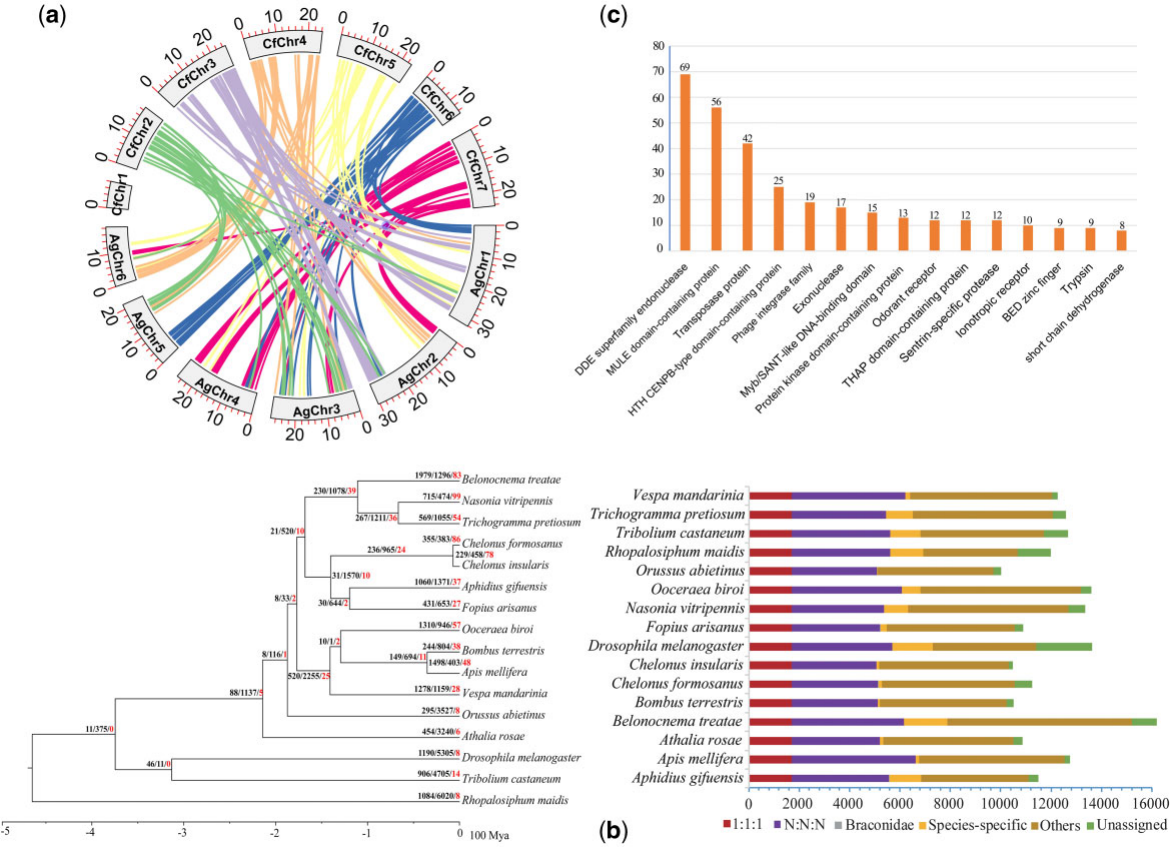
Chromosomal synteny, phylogeny, and gene family evolution of *Chelonus formosanus*. (*a*) Chromosomal synteny between *Chelonus formosanus* (CfChr) and *Aphidius gifuensis* (AgChr) genomes. (*b*) In the phylogeny, the node values on the tree represent the number of expanded, contracted, and rapidly evolving families for each clade or species. Statistics of orthology inference result: “1:1:1” indicates single-copy orthologs; “N:N:N” indicates multicopy orthologs; “Braconidae” indicates orthologs are specific to Braconidae; “Others” indicates unclassified orthologs; “Unassigned” indicates orthologs that cannot be assigned into any of the orthogroups. (*c*) The top 15 significantly expanded gene families with numbers of genes in each family shown above each bar.

We obtained a total of 35.4 Gb PacBio long (∼150 × coverage) and 36.43 Gb Illumina short reads. The average length and N50 length of the long reads were 15.23 and 17.94 kb, respectively. The kmer analysis predicted genome size being 139.59 Mb, and it also indicated no significant heterozygosity and approximately 18 Mb (12.95%) repetitive sequences of the genome (supplementary fig. S2, Supplementary Material online). The genome assembly size, GC content, and Benchmarking University Single-Copy Orthologs (BUSCO) assessment results are comparable to the genome assembly of the closely related species *C. insularis* (table 1). However, our genome assembly was more complete with smaller number of scaffolds and more contiguous with fewer gaps compared with *C. insularis*. The mapping-back rates from the Illumina DNA and RNA sequences as well as the PacBio raw reads were 98.29%, 96.92%, and 96.02%, respectively. Overall, our *C. formosanus* genome scaffolds have recovered most of sequencing reads and is suitable for further analysis. According to the long-range linked reads from Hi-C data, we assigned 138.84 Mb of the assembly to the seven pseudochromosomes (supplementary fig. S2, Supplementary Material online). A chromosomal synteny analysis between the *C. formosanus* and *Aphidius gifuensis* Ashmaed, 1906 chromosomes showed limited level of conservation. We also noticed that there was no indication of conservation between the chromosome one of *C. formosanus* and the *A. gifuensis* genome (fig. 1*a*). To investigate this further, more chromosomal conservation analysis with other closely related species may be applied when the chromosome-level genome assemblies of those species became available.

**Table 1:** Genome Assembly Statistics of *Chelonus formosanus* Compared with *Chelonus insularis*

	*Chelonus formosanus*	*Chelonus insularis*
Genome assembly		
Assembly size (Mb)	139.590	135.730
Number of scaffolds/contigs	26/106	455/457
Longest scaffold/contig (Mb)	24.95/15.194	4.699/4.699
N50 scaffold/contig length (Mb)	24.159/5.591	1.163/1.163
GC (%)	30.36	30.53
Gaps (%)	0.006	0.043
BUSCO completeness (%)	99.0	99.1

### Genome Annotation

There were 22.25% of the assembly annotated as repetitive sequences. This is over 4% larger than the prediction form the kmer analysis, which is likely due to a better capture of repetitive sequences from long-read assembly. Except for the unclassified repeats (8.62%), DNA elements were the most abundant repeat type (6.19%) in the assembly, followed by the long-terminal repeat elements (LTR; 3.42%), simple repeats (1.79%), and long-interspersed nuclear elements (0.91%) (supplementary table S1, Supplementary Material online). We also predicted 324 noncoding RNAs (ncRNAs) including 38 micro-RNAs (miRNAs), 69 ribosomal RNAs (rRNAs), 28 small nuclear RNAs (snRNAs), 108 transfer RNAs (tRNAs), and 44 others (supplementary table S2, Supplementary Material online). The annotated snRNAs include 14 spliceosomal RNAs (U1, U2, U4, U5, U6, U11), three minor spliceosomal RNAs (U4atac, U6atac, U12), six C/D box snoRNAs, and four H/ACA box snoRNA.

A total of 4.42-Gb RNA-Seq reads were imported into the gene prediction program MAKER as biological evidence for the protein-coding gene prediction. The MAKER process predicted 11,242 protein-coding gene models, which was comparable to that of *C. insularis* (11,574). The average gene and protein-coding region lengths were 4,350 and 1,593 bp, respectively. The average exon length and the average number of exons per gene were 355.13 bp and 5.73, respectively. The average intron length was 522.85 bp. The BUSCO assessment result showed 98.6% complete genes were captured, and 0.4% and 1.0% of the genes were fragmented and missing, respectively. There were 9,019 (80.23%) protein-coding genes identified with protein domains, which were then assigned with 7,822 GO terms, 6,157 KEGG KO terms, 2,020 enzyme codes, 3,576 KEGG pathways, 2,364 Reactome pathways, and 9,071 COG categories (supplementary figs. S3 and S4, Supplementary Material online).

### Phylogenomics and Gene Family Evolution

A total of 184,525 (94.9%) genes obtained from 16 species were clustered into 13,537 gene families. There are 4,640 gene families with all the species sequences present including 1,702 single-copy and 2,938 multicopy gene families. Among the 11,242 annotated *C. formosanus* genes, 10,556 were clustered into 8,937 families (fig. 1*b*). There were 68 genes present in 48 families that are specific to *C. formosanus* (fig. 1*b*). A phylogenetic tree with the bootstrap support value of 100/100 was reconstructed based on the 1,581 genes after 121 single-copy genes were removed (fig. 1*b*). The topology of this phylogeny shows consistency with the previous phylogenetic tree constructed by Peters et al. (2017). For example, parasitoid Apocrita belongs to a monophyletic group and forms a sister group to Orussoidea (*Orussus abietinus*), and Aculeata, Chalcidoidea, and Ichonoidea are all monophyletic groups (Gauthier et al. 2021). As expected, our analysis showed *C. formosanus* was closely clustered with *C. insularis* (fig. 1*b*) and our calculation indicated the two species diverged approximately 6 Ma.

We identified 355 expanded (58 significantly expanded) and 383 contracted (28 significantly contracted) gene families from the *C. formosanus* annotated gene models (fig. 1*c*). There was a rapid expansion of the allelopathic families (odorant receptors and ionotropic receptors) and a digestion-related family (trypsin) (fig. 1*c*). The gene ontology enrichment results also show a rapid expansion of the gene families belonging to the GO terms of olfactory receptor (OR), odorant binding, and sensory perception of smell (supplementary fig. S4, Supplementary Material online). ORs play an important role in locating host during parasitic process (Gauthier et al. 2021). The expansion of these genes we found in the *C. formosanus* genome might explain its wide range of insect hosts.

## Materials and Methods

### Sample Collection and Sequencing

*Chelonus formosanus* specimens were collected in June 2020 within the Guilinyang Economic Development Zone, Haikou City, Hainan Province, China (20.0521°N, 110.2067°E) and then reared with *Spodoptera frugiperda* for more than five generations under the laboratory conditions of 26 ± 1 °C, 70 ± 5% RH, and a photoperiod of 14:10 (L:D) h. Male adults were used for genome sequencing: four individuals for Illumina, 20 for PacBio, and five for Hi-C. Four males were used for RNA-Seq (supplementary fig. S1, Supplementary Material online).

The high-quality DNA was extracted using the QIAGEN DNeasy Blood & Tissue kit. For the PacBio sequencing, a 20-kb insert size library was constructed using the SMRTbell Template Prep Kit 2.0. For Illumina DNA sequencing, a library with an insert size of 350 bp was constructed using the TruSeq DNA PCR-free kit. The Hi-C library construction (restriction enzyme: *Mbo*I) was performed by Frasergen Co., Ltd (Wuhan, China). RNA was extracted using the TRIzolTM Reagent kit and the RNA libraries were constructed using the TruSeq RNA v2 kit. The Illumina and PacBio sequencing were performed on NovaSeq 6000 and PacBio Sequel II, respectively, at the Beijing Berry Genomics Co., Ltd (Beijing, China).

### Genome Assembly

The quality control of the Illumina short reads was performed using BBTools suite v38.49 (Bushnell 2014): the script “Clumpify.sh” was used to remove duplicated sequences; “bbduk.sh” was used for trimming sites with base quality scores below 20 (>Q20) and poly-A/G/C ends with their lengths being more than 10 bp, filtering sequences with lengths below 15 bp, and correcting bases according to the sequence overlap regions (qtrim=rl trimq = 20 minlen = 15 ecco=tmaxns = 5 trimpolya = 10 trimpolyg = 10 trimpolyc = 10). The k-mer frequency was calculated using the “khist.sh” script from the BBTools suite (kmer: 21). A genomic survey based on the k-mer distribution frequency was performed using Genomescope v2.0 (Vurture et al. 2017) with the parameters of “-k 21 -p 2 -m 10,000.”

The long-read assembler Flye v2.8.1 (Kolmogorov et al. 2019) was used to generate a preliminary genome assembly with the parameters “-i 2 -m 1,000” (two rounds of long-read polishing with a minimum overlap length of 1,000 bases between sequences). The Illumina reads were then aligned to the preliminary assembly using Minimap2 v2.17 (Li 2018) with default parameters and the alignments were used for the two consecutive rounds of short-read polishing with NextPolish v1.3.0 (Hu et al. 2020). The haplotigs and overlaps in the genome assembly were filtered based on the read depth using Purgedups v1.0.1 (Guan et al. 2020) with the minimum alignment score of 70 (-a 70). The remaining contigs were then assigned to pseudochromosomes based on the contact information from the read alignments of the Hi-C data: first, the raw Hi-C reads were quality-assessed and then removed unusable reads using Juicer v1.6.2 (Durand et al. 2016); second, the pseudochromosomal assignment was performed using 3D-DNA v180922 (Dudchenko et al. 2017); third, the assignment errors were corrected using Juicebox v1.11.08 based on the Hi-C contact maps (Durand et al. 2016). The contaminant sequences were removed using BLAST+ (BlastN) v2.7.1 (Camacho et al. 2009) based on the homological search against the NCBI nucleotide (nt; downloaded on 31^st^ of December 2020) and UniVec databases. Genome completeness was assessed using BUSCO v3.0.2 pipeline (Waterhouse et al. 2018) by searching against the database of insect_odb10 database (*n* = 1,367).

To construct a chromosomal synteny between the *C. formosanus* and *A. gifuensis* pseudochromosomes, A BlastP-like alignment method was performed using Mmseq2 v11-e1a1c with default parameters for aligning protein sequences. The generated “all.blast” file and the integrated “all.gff” file were imported in MCScanX to perform collinearity analysis. A circus plot was created using Tbtools v1.0692 (Chen et al. 2020).

### Genome Annotation

The genome assembly was annotated for repetitive sequences, protein-coding genes, and ncRNAs. To annotate repeats, a de novo repeat library was constructed using RepeatModeler v2.0.1 (Flynn et al. 2020) with the LTR search process (-LTRStruct). It was then combined with Dfam3.3 (Hubley et al. 2016) and the RepBase-20181026 databases (Bao et al. 2015) to form a custom library, which was used as input for RepeatMasker v4.0.9 (Smit et al. 2013–2015) to search for repeats and generate a repeat-masked assembly. To annotate ncRNAs, the rRNAs, snRNAs, and miRNAs were identified based on the alignment with the Rfam library using Infernal v1.1.2 (Nawrocki and Eddy 2013), and the tRNAs were predicted using tRNAscan-SE v2.0.6 (Chan and Lowe 2019) and then filtered low-confident sequences using the “EukHighConfidenceFilter” script.

Protein-coding genes were predicted using MAKER v.3.01.03 (Holt and Yandell 2011), with three supporting evidence files produced from other programs: 1) Ab initio predicted genes generated from BRAKER v2.1.5 (Hoff et al. 2016), which trains Augustus v3.3.2 (Stanke et al. 2004) and GeneMark-ES/ET/EP 4.483.60lic (Lomsadze et al. 2005) based on the RNA-Seq alignments generated from HISAT2 v2.2.0 (Kim et al. 2019) and the OrthoDB10 v1 protein database (Kriventseva et al. 2019) to improve prediction accuracy; 2) Transcript sequences assembled using StringTie v2.1.4 (Kovaka et al. 2019) from the RNA-Seq alignments generated by HISAT2; 3) Protein sequences of closely related species [*Drosophila melanogaster* Meigen, 1830, *Tribolium castaneum* (Herbst, 1797), *Apis mellifera* L., 1761, *Bombus terrestris* (L. 1758), *Nasonia vitripennis* (Walker, 1836), and *Bombyx mori* L., 1758] downloaded from NCBI. Gene functions were annotated using Diamond v0.9.24 (Buchfink et al. 2015) with the sensitive mode (–more-sensitive -e 1e-5) to search against UniProtKB and using InterProScan 5.41–78.0 (Finn et al. 2017) to search against Pfam (El-Gebali et al. 2019), Smart (Letunic and Bork 2018), Gene3D (Lewis et al. 2018), Superfamily (Wilson et al. 2009), and CDD (Marchler-Bauer et al. 2017) databases. The eggnog-mapper v2.0.1 (Huerta-Cepas et al. 2017) was also used to search against the eggnog v5.0 (Huerta-Cepas et al. 2019) database to predict conserved sequences and domains, GO terms, and protein pathways (KEGG, Reactome).

### Phylogenomics and Gene Family Evolution

There were 16 species selected for the orthology inference using OrthoFinder, which used Diamond v2.3.8 for rapid protein sequence aligning (Emms and Kelly 2019). These species included *Rhopalosiphum maidis* (Fitch, 1856) from Heteroptera, *Tribolium castaneum* (Herbst, 1797) from Coleoptera, *Drosophila melanogaster* Meigen, 1830 from Diptera, *Athalia rosae* (Linnaeus, 1758) and *Orussus abietinus* (Scopoli, 1763) from Symphyta, *Apis mellifera*, *B. terrestris*, *Ooceraea biroi* (Forel, 1907) and *Vespa mandarinia* Smith, 1852 from Aculeata, and seven parasitic wasp species [*Aphidius gifuensis* Ashmaed, 1906, *Belonocnema treatae* Mayr, 1881, *C. formosanus*, *C. insularis*, *Fopius arisanus* (Sonan, 1932), *N. vitripennis*, and *Trichogramma pretiosum* Riley, 1879]. A species phylogeny was constructed using 1,702 single-copy orthologs as following: first, regions of homologous sequences were aligned using MAFFT v7.394 (Katoh and Standley 2013) with the option of L-INS-I; second, the unreliable regions from the alignments were trimmed using BMGE v1.12 (Criscuolo and Gribaldo 2010) with the parameter of “-m BLOSUM90-h 0.4.”; third, the modified alignments were combined to a supermatrix using FAScoCAT-G v1.04. Phylogenetic tree construction was performed using IQ-TREE v2.0-rc1 (Minh et al. 2020) with the parameters of “-symtest-remove-bad -symtest -pval 0.10” to remove sequences that did not meet the substitution, reversible, or homogeneous hypotheses. To reduce computational resources, the model type was limited to LG (-m MFP –mset LG –msub nuclear –rclusterf 10). The bootstrap values were calculated using ultrafast bootstrap and the SH-aLRT algorithm (-B 1,000 –alrt 1,000). The clock dating of species divergence was performed using MCMCTree (clock = 2, BDparas = 1 1 0.1, kappa_gamma = 6 2, alpha_gamma = 1 1, rgene_gamma = 2 20 1, sigma2_gamma = 1 10 1) from the PAML v4.9j package (Yang 2007). The evidence for the fossil calibration points was obtained from the PBDB database (https://www.paleobiodb.org/navigator/, last accessed July 11, 2021): Trichoptera, (3.114–3.146 Ma); Hymenoptera, (2.056–2.12 Ma); Aculeata, (1.402–1.45 Ma); Chalcidoidea, (0.935–0.996 Ma); and Ichonoidea, (1.402–1.45 Ma). The estimation of gene family expansion and contraction was performed using CAFÉ v4.2.1 (Han et al. 2013) with the *p* parameter of 0.01. The R package “clustering profiler” v3.10.1 (Yu et al. 2012) with default parameters was used to analyze and visualize the enriched GO ontology terms and KEGG pathways of the significantly expanded gene families. 

## Supplementary Material

Supplementary data are available at *Genome Biology and Evolution* online.

## Supplementary Material

evac006_Supplementary_Data
