# Supplementary figures and images for: A Chromosome-Level Genome Assembly of the Parasitic Wasp Chelonus formosanus Sonan 1932 (Hymenoptera: Braconidae)
Source: Genome Biol Evol. 2022 Jan 28;14(1):evac006. doi: 10.1093/gbe/evac006 (PMC8808538; doi:10.1093/gbe/evac006)

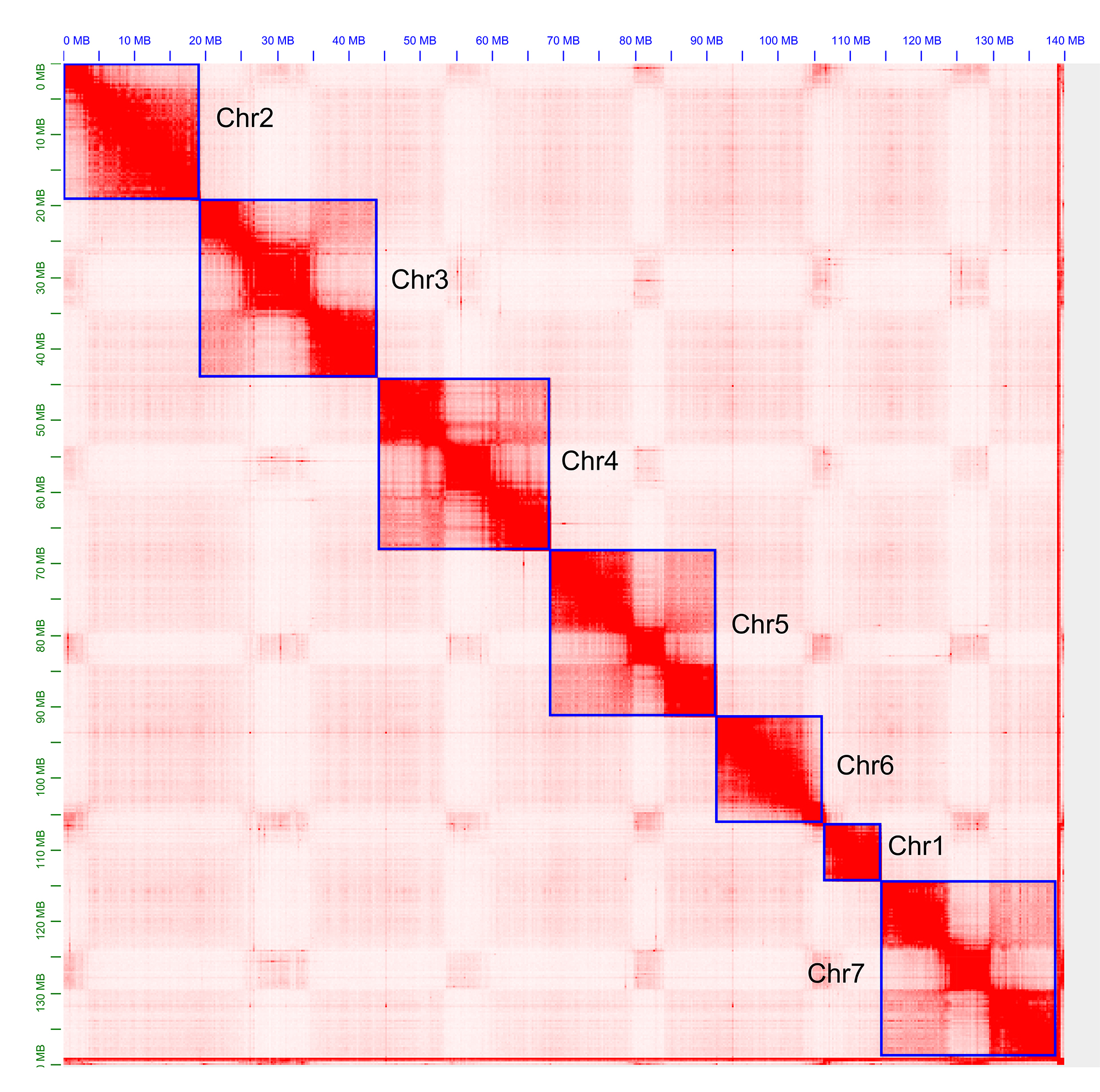

Supplement: evac006_Supplementary_Data [file evac006_supplementary_data.zip › Figure S2.jpg]

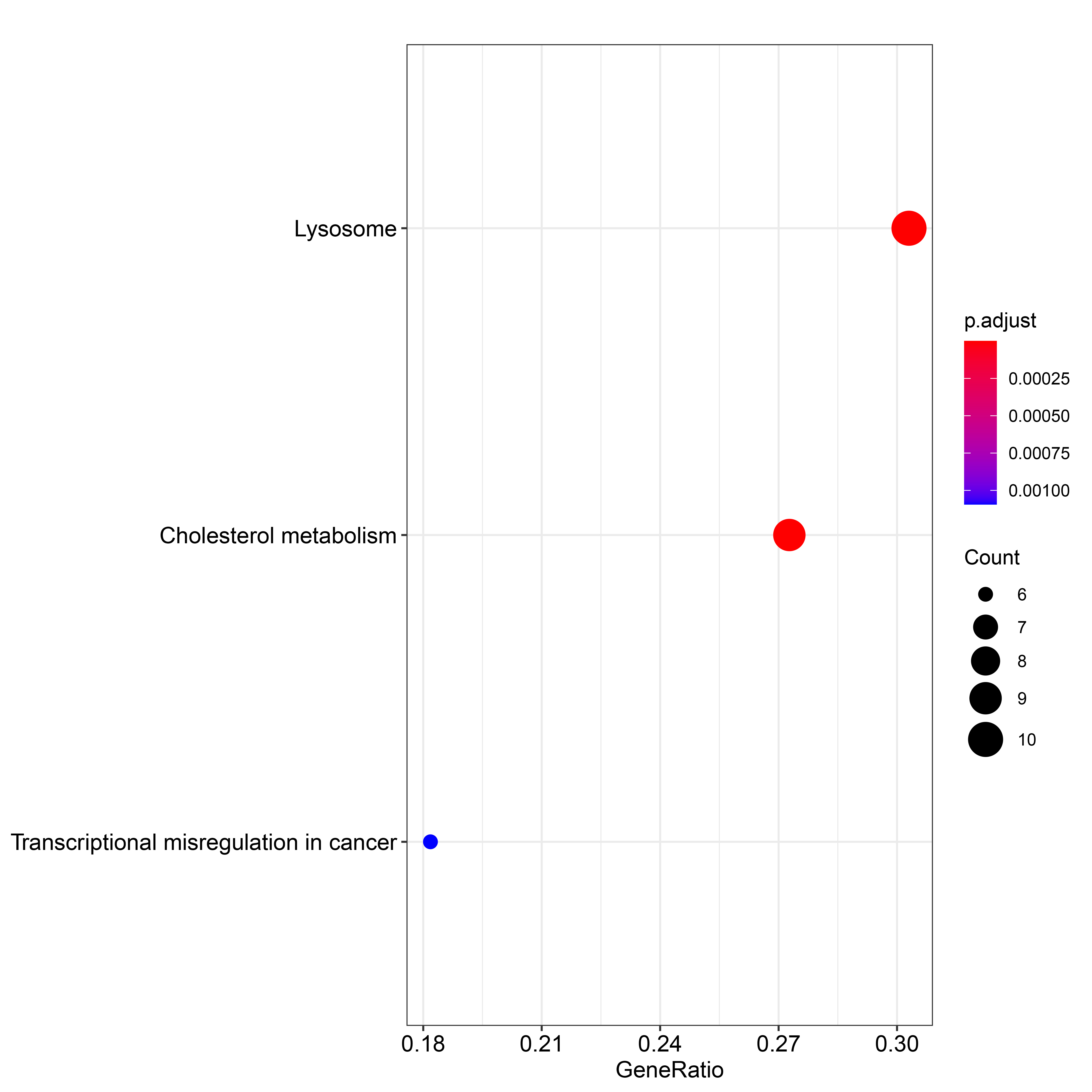

Supplement: evac006_Supplementary_Data [file evac006_supplementary_data.zip › Figure S3.jpg]

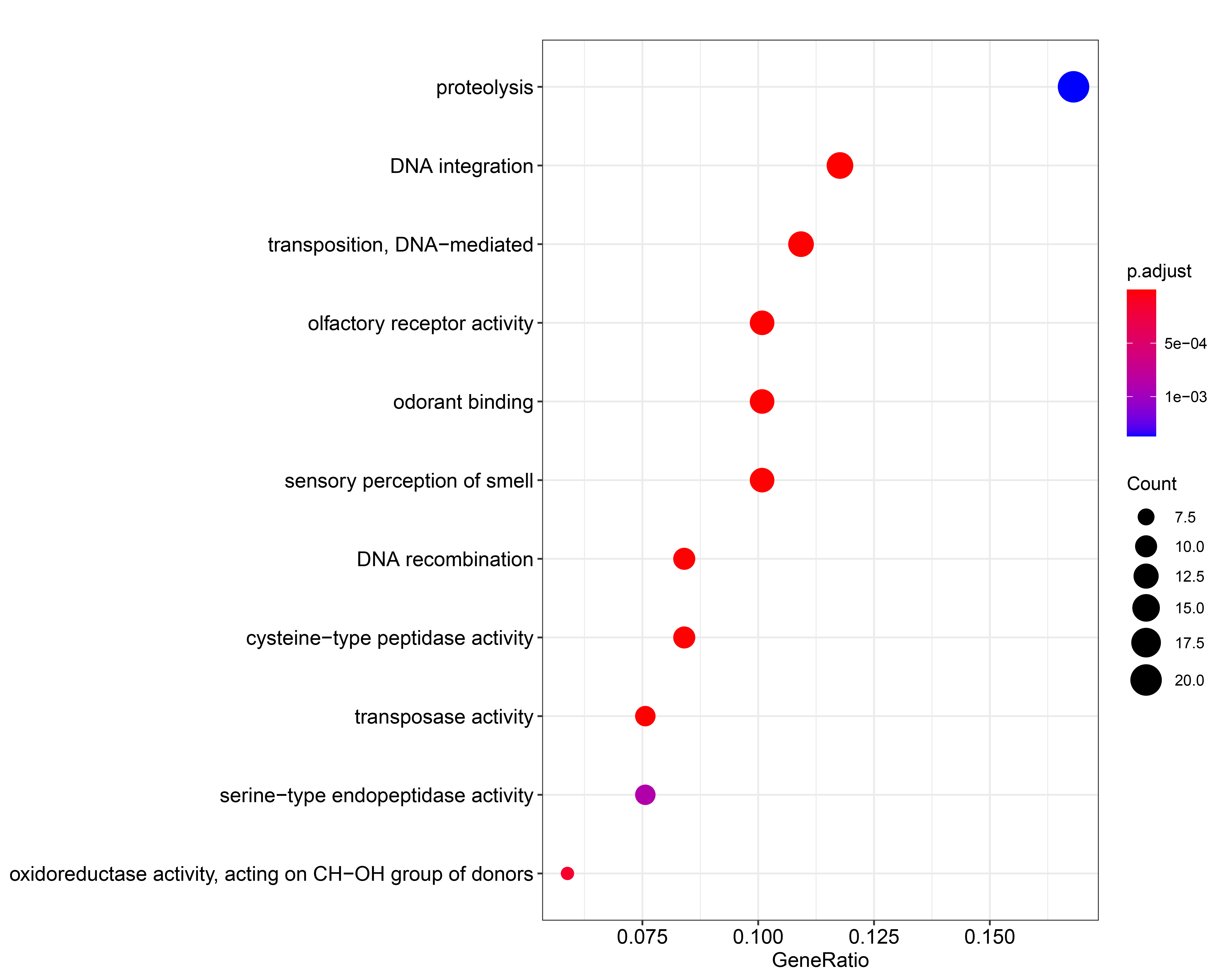

Supplement: evac006_Supplementary_Data [file evac006_supplementary_data.zip › Figure S4.jpg]

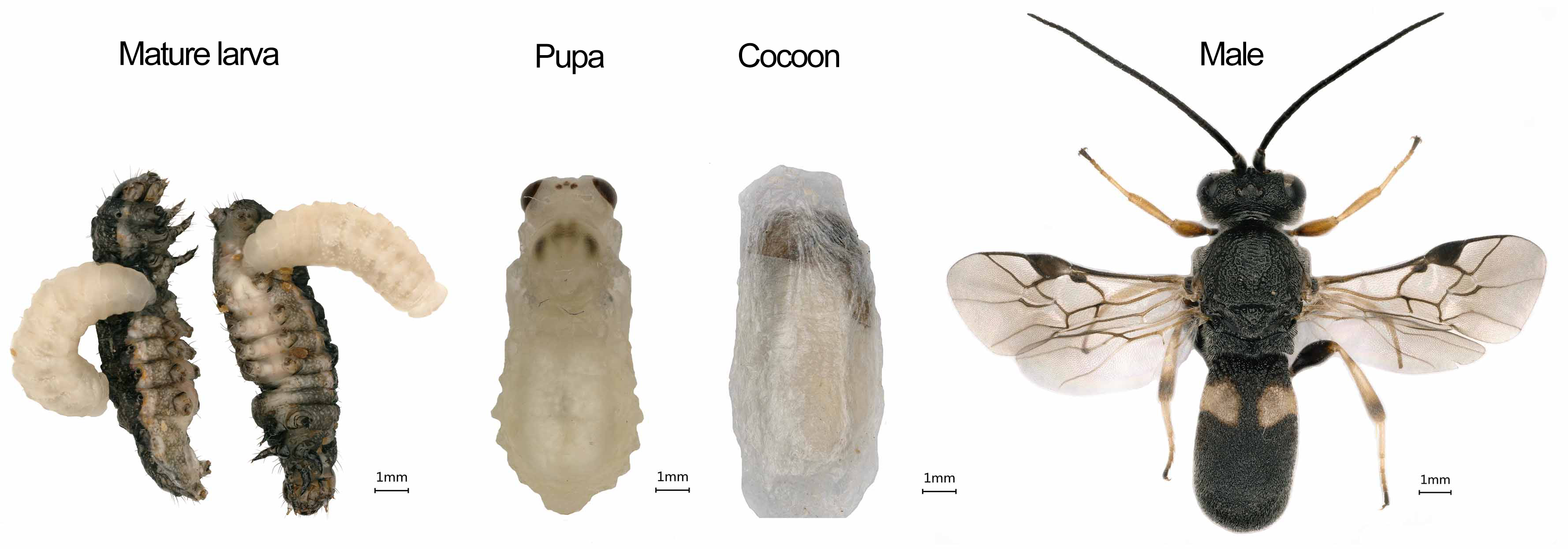

Supplement: evac006_Supplementary_Data [file evac006_supplementary_data.zip › Figure S1.jpg]
